# Biodiversity of *Trichoderma* species of healthy and *Fusarium* wilt-infected banana rhizosphere soils in Tenerife (Canary Islands, Spain)

**DOI:** 10.3389/fmicb.2024.1376602

**Published:** 2024-05-10

**Authors:** Raquel Correa-Delgado, Patricia Brito-López, María C. Jaizme Vega, Federico Laich

**Affiliations:** Unidad de Protección Vegetal, Instituto Canario de Investigaciones Agrarias, Valle de Guerra, Santa Cruz de Tenerife, Canary Islands, Spain

**Keywords:** banana *Fusarium* wilt, Panama disease, *Fusarium oxysporum* f. sp. *cubense* subtropical race 4, *Trichoderma*, biodiversity, soil rhizosphere, biological control agent, *Musa acuminata*

## Abstract

Banana (*Musa acuminata*) is the most important crop in the Canary Islands (38.9% of the total cultivated area). The main pathogen affecting this crop is the soil fungal *Fusarium oxysporum* f. sp. *cubense* subtropical race 4 (*Foc*-STR4), for which there is no effective control method under field conditions. Therefore, the use of native biological control agents may be an effective and sustainable alternative. This study aims to: (i) investigate the diversity and distribution of *Trichoderma* species in the rhizosphere of different banana agroecosystems affected by *Foc*-STR4 in Tenerife (the island with the greatest bioclimatic diversity and cultivated area), (ii) develop and preserve a culture collection of native *Trichoderma* species, and (iii) evaluate the influence of soil chemical properties on the *Trichoderma* community. A total of 131 *Trichoderma* isolates were obtained from 84 soil samples collected from 14 farms located in different agroecosystems on the northern (cooler and wetter) and southern (warmer and drier) slopes of Tenerife. Ten *Trichoderma* species, including *T. afroharzianum, T. asperellum, T. atrobrunneum, T. gamsii, T. guizhouense, T. hamatum, T. harzianum, T. hirsutum, T. longibrachiatum*, and *T. virens*, and two putative novel species, named *T*. aff. *harzianum* and *T*. aff. *hortense*, were identified based on the *tef1-α* sequences. *Trichoderma virens* (35.89% relative abundance) and *T.* aff. *harzianum* (27.48%) were the most abundant and dominant species on both slopes, while other species were observed only on one slope (north or south). Biodiversity indices (Margalef, Shannon, Simpson, and Pielou) showed that species diversity and evenness were highest in the healthy soils of the northern slope. The Spearman analysis showed significant correlations between *Trichoderma* species and soil chemistry parameters (mainly with phosphorus and soil pH). To the best of our knowledge, six species are reported for the first time in the Canary Islands (*T. afroharzianum, T. asperellum, T. atrobrunneum, T. guizhouense, T. hamatum, T. hirsutum*) and in the rhizosphere of banana soils (*T. afroharzianum, T. atrobrunneum, T. gamsii, T. guizhouense, T. hirsutum*, *T. virens*). This study provides essential information on the diversity/distribution of native *Trichoderma* species for the benefit of future applications in the control of *Foc*-STR4.

## Introduction

The Canary Island archipelago comprises eight islands located in the subtropical region of the Atlantic Ocean near the Tropic of Cancer, off the African coast of Western Sahara (between 27°37′-29°25′N and 13°20′-18°10′W). Its climate is characterized by low annual thermal variations, mainly due to the influence of cold ocean currents and trade winds from the NE (cold and humid) that blow for most of the year (Carracedo et al., 2002). The Canary Islands are one of the most biodiverse areas in the European Union and one of the most outstanding in the world for its endemism. This region contains half of Spain’s endemic flora and provides the right conditions for the cultivation of a wide variety of subtropical and tropical species (Madruga et al., 2016). Banana (*Musa acuminata*) is the most important crop in the Canary Islands. It covers a total area of 8,891 ha (38.9% of the cultivated area of the whole archipelago) and provides 63% of the total banana production in Europe (Food and Agriculture Organization of the United Nations, 2022; Instituto Canario de Estadística [ISTAC], 2022). Due to the commercial importance of the crop, since 2013, it has been included in the Register of Protected Designations of Origin (PDO) and Protected Geographical Indications (PGI) (EUR-Lex, 2013).

Various pests and diseases affect banana crops in subtropical areas. Among these, *Fusarium* wilt of musaceae, also known as Panama disease is worth highlighting. This disease is caused by the soil fungus *Fusarium oxysporum* f. sp. *cubense* (*Foc*) and is one of the most destructive diseases for the musaceae (Ploetz, 2015a). Tropical race 4 (*Foc*-TR4) is the most pathogenic among the major commercial cultivars of the Cavendish subgroup (AAA) (Ploetz, 2006). In the Canary Islands, studies carried out at the Department of Plant Protection of the Canary Institute of Agricultural Research (ICIA)^1^ from the 1980s to the present have shown that the causal agent of Panama disease is the subtropical race 4 of *Foc* (*Foc*-STR4) and that *Foc*-TR4 has not been detected. Perera-González et al. (2023) showed that 23% of banana farms on Tenerife had plants with *Foc* symptoms (yellowing of leaves, drying of leaves, and xylem necrosis on corm and pseudostem). Nevertheless, the incidence of the disease is generally low (3.4% of plants on affected farms present symptoms of the disease), and these plants are usually found in places with poor drainage, acid pH, soil compaction, excessive humidity, and/or shady areas. In other (minority) cases, the incidence of the disease is higher (49.1% of plants with symptoms) and leads to abandonment or change in crops.

A large number of publications describe different alternatives to control the pathogen, however, several reasons make control difficult (Ploetz, 2015b; Dita et al., 2018; Siamak and Zheng, 2018). Among them are: (a) the ability of the pathogen to survive in the soil for long periods of time (more than 40 years) (Buddenhagen, 2009); (b) the spread of the pathogen through contaminated materials (plants, tools, farm equipment, irrigation water, etc.); (c) the endophytic properties of the fungus, which protect it from the action of contact fungicides or non-endophyte biological control agents (Bubici et al., 2019); (d) the lack of rotation in most commercial crops of intensive production (monoculture) cause the pathogen to multiply in a continuous cycle, increasing the inoculum in the soil over time (Dita et al., 2018). Considering these factors, probably, the most effective alternative for its control could be the use of resistant cultivars, although the appearance of new pathogenic variants of the fungus can overcome this resistance (Ploetz, 2015b; Dita et al., 2018). Nevertheless, the key measures to control the disease and prevent the spread of the pathogen in those regions free of the fungus or with low infestation levels are pathogen exclusion, contingency plans and destroying infected materials (Ploetz, 2015b; Dita et al., 2018).

Other management alternatives aimed at improving soil health are mainly based on the application of appropriate agronomic practices, the use of cover crops or the application of organic amendments and biological control agents (BCA) (Dita et al., 2018). In this sense, soil microorganisms have a fundamental role in plant health and protection, especially those that colonize the rhizosphere zone, by enhancing nutrient uptake, disease resistance, plant defense response, and tolerance to various biotic and abiotic stresses (Raaijmakers et al., 2009; Liu et al., 2021). A well-researched example is the genus *Trichoderma* (syn. Hypocrea, Hypocreales), which consist of more than 400 species (Cai and Druzhinina, 2021) divided into three representative groups, *Harzianum*, *Trichoderma*, and *Longibrachiatum* (Kubicek et al., 2019), known as linages (Gutiérrez et al., 2021). *Trichoderma* is a cosmopolitan and opportunistic filamentous fungus and a ubiquitous colonizer in almost all environments, commonly found in agriculture soils (Woo et al., 2023). Of particular interest in this genus are the processes involved in the biological control of plant diseases, with direct action on plant pathogens and indirect mechanisms through the induction of local and systemic plant defenses (Harman et al., 2004). Due to these characteristics, *Trichoderma* has remained a notable BCA and has become a popular protagonist as the key component of plant biostimulants, bioprotectants, biofertilizers, and soil amendments (Woo et al., 2023).

In relation to the biological control of *Foc*, a wide variety of scientific articles describe the biocontrol potential of *Trichoderma* against this pathogen (mainly on *Foc*-TR4) (Bubici et al., 2019; Izzati et al., 2019; Damodaran et al., 2020). However, there are few studies that evaluate *Trichoderma* isolates obtained from banana soils or plants (Thangavelu et al., 2004; Thangavelu and Mustaffa, 2010; Caballero et al., 2013; Galarza et al., 2015; Thangavelu and Gopi, 2015; Chaves et al., 2016; Taribuka et al., 2017; Damodaran et al., 2020; Olowe et al., 2022). In many cases, the reduced efficacy of BCAs under field conditions could be due to their inability to grow under local biotic and abiotic environmental conditions. For this reason, it is extremely important to know the natural microbial biodiversity in the different ecosystems and to have a wide collection of native strains adapted to the specific agroclimatic conditions of the crop where it is to be applied.

The main purpose of this study was to collect a representative number of rhizosphere soil samples from healthy and *Foc*-STR4-affected banana plants in different agroecosystems on Tenerife, in order to: (i) describe the diversity and distribution of *Trichoderma* species in different agroecosystems, (ii) develop and preserve a culture collection of native *Trichoderma* species, and (iii) evaluate the influence of soil chemical properties on *Trichoderma* community composition. The practical importance of this study is to provide an initial knowledge base about the diversity and distribution of *Trichoderma* species in different banana ecosystems of Tenerife, and to preserve a culture collection of native *Trichoderma* as a reservoir of potential beneficial microorganisms to develop sustainable agro-biotechnological alternatives. Indeed, it is important to highlight the role of the microbial culture collection as an essential source for the selection of potential BCA against *Foc*-STR4 or plant growth promoting microorganisms (PGPM) naturally adapted to the agroclimatic conditions of banana crops in the Canary Islands.

This is the first work that describes the *Trichoderma* community in the rhizosphere soil of banana plants with and without symptoms of Panama disease in the different bioclimatic conditions and growing areas of the island of Tenerife. In addition, six species of *Trichoderma* are reported for the first time in the Canary Islands (*T. afroharzianum, T. asperellum, T. atrobrunneum, T. guizhouense, T. hamatum*, and *T. hirsutum*) and six species that have not been previously reported associated with banana rhizosphere (*T. afroharzianum, T. atrobrunneum, T. gamsii, T. guizhouense, T. hirsutum*, and *T. virens*). Furthermore, we detected two groups of isolates, which we identified as *T.* aff. *harzianum* and *T.* aff. *hortense*, corresponding to putative novel endemic species to be described in the near future.

## Materials and methods

### Crop distribution and agroclimatic characteristics of Tenerife

Approximately 9,000 farmers cultivate bananas in the Canary Islands on an area of about 8,891.2 ha. The island with the largest cultivated area is Tenerife (4,002.9 ha), followed by La Palma (2,727.6 ha), and Gran Canaria (1,936.6 ha) (Instituto Canario de Estadística [ISTAC], 2022). In addition, Tenerife (situated near the center of the archipelago) is the largest (2,034 km^2^) and highest island (3,718 m.a.s.l.) of the archipelago and has the largest number of bioclimatic belts (26): from the hyperarid desertic inframediterranean in the southern zone of the coast to the dry pluviseasonal oromediterranean at higher altitudes (3,718 m.a.s.l., Mount Teide) (Del-Arco et al., 2006).

In Tenerife, banana crops are distributed around almost the entire perimeter of the island, mainly at altitudes below 300 m.a.s.l. The main environmental difference is observed when comparing the northern and southern slopes. The northern slope is wetter and cooler (due to the trade winds and annual rainfall of up to 500 mm), while the southern slope is drier and warmer (exposed to heatwaves from the Sahara and with less than 200 mm annual rainfall) (Supplementary Table 1). These differences affect crop development, with significant differences between slopes in terms of leaf emission and bunch emergence intervals because of temperature (Galán-Sauco et al., 1984). Also, on both slopes, altitude (related to temperature) has a significant effect. As altitude increases, the emergence interval becomes longer. The soil type in both slope is Andisols (Tejedor et al., 2009).

### Characteristics of the sampling points and experimental design

The sampling points were selected considering the following factors: (a) the agroclimatic characteristics, (b) the distribution of banana production areas, and (c) the distribution of *Foc*-STR4-affected banana plants. As a reference for the distribution of Panama disease (*Foc*-STR4) in Tenerife, we used the analysis of a previous work in which 100 farms spread randomly and proportionally over the cultivated area in each of the banana production zones were studied (Perera-González et al., 2023). For this purpose, a grid (500 m × 500 m) was implemented over the entire cultivated area of banana crops and several squares proportional to the cultivated area of each zone were randomly selected. In each square (approximately one per 40 ha), the largest farm was selected and an assessment of Panama disease was carried out (Perera-González et al., 2023). Taking into account the results of this study and the factors mentioned above, 14 farms (8 on the north slope and 6 on the south) with plants affected by *Foc*-STR4 distributed representatively in each production zone were selected. All selected farms had a history of banana monoculture of more than 15 years. The geographical, bioclimatic data, and the cropping history of each farm are given in Table 1.

**TABLE 1:** Geographical, bioclimatic data, and cropping history of the 14 sampling farms in Tenerife (Canary Islands).

Farm code	Municipality	Location	Slope	Thermotype^a^	Bioclimatic belt^a^	Coordinates	Years of planting^b^	Altitude (m.a.s.l.)
BN47	Buenavista del Norte	Las Toscas	North	Inframediterranean	Lower-semiarid xeric inframediterranean	28°22′ 13.52″N 16°50′ 37.76″W	35	124.69
IV72	Icod de Los Vinos	Las Granaderas	North	Inframediterranean	Lower-semiarid xeric inframediterranean	28°23′ 26.83″N 16°32′ 06.74″W	15	164.02
IV37	Icod de los Vinos	Valois	North	Inframediterranean	Lower-semiarid xeric inframediterranean	28°22′ 32.54″N 16°43′ 31.47″W	20	109.71
VO78	La Orotava	El Rincón	North	Inframediterranean	Lower-semiarid xeric inframediterranean	28°25′ 09.73″N 16°30′ 34.49″W	50–60	105.59
VO64	La Orotava	San Miguel	North	Inframediterranean	Upper-semiarid xeric inframediterranean	28°22′ 16.60″N 16°43′ 27.16″W	70	278.12
BN4	Los Silos	Hoya Matos	North	Inframediterranean	Lower-semiarid xeric inframediterranean	28°22′ 40.08″N 16°49′ 16.25″W	25	47.71
TV26	San Cristóbal de La Laguna	La Cardonera (Tejina)	North	Inframediterranean	Lower-semiarid xeric inframediterranean	28°32′ 29.91″N 16°21′ 55.07″W	40	103.56
PH1	San Cristóbal de La Laguna	Punta Hidalgo	North	Inframediterranean	Lower-semiarid xeric inframediterranean	28°34′ 13.98″N 16°19′ 08.72″W	50–60	73.21
AD46	Adeje	La Tiñosa (Barranco Las Moradas)	South	Inframediterranean	Arid desertic inframediterranen	28°09′ 04.23″N 16°46′53.99″W	15	177.33
AR51	Arona	Llanos de Guargacho	South	Inframediterranean	Arid desertic inframediterranen	28°02′ 23.23″N 16°38′ 23.56″W	15	101.03
AR68	Arona	Buzanada	South	Inframediterranean	Arid desertic inframediterranen	28°04′ 29.14″N 16°39′ 17.31″W	40	304.83
CPE	Guía de Isora	Cueva del Polvo	South	Inframediterranean	Arid desertic inframediterranen	28°13′ 47.11″N 16°49′ 58.73″W	30	104.86
GUI	Güímar	Hoya del Cerco	South	Inframediterranean	Arid desertic inframediterranen	28°18′ 39.40″N 16°23′ 03.31″W	25	115.03
SM109	San Miguel de Abona	Montaña de Los Gorones	South	Inframediterranean	Arid desertic inframediterranen	28°03′ 11.28″N 16°37′ 54.31″W	35	159.03

^a^ Del-Arco et al. (2006).

^b^Years of the farm with banana crop (monoculture).

### Soil and plant sample collection

Six plants per farm were selected: three plants with visible symptoms of *Foc*-STR4 disease and three asymptomatic plants. Plants with symptoms were selected as far apart as possible. The selection of asymptomatic plants was carried out on specimens with the same growing conditions and phenological state as the plants with symptoms, at 5–10 m from them.

Rhizosphere soil samples were collected around each plant (50 cm from the pseudostem) at four opposite points, discarding the upper plant remains and using a 45-mm diameter soil auger at 0–20 cm depth. Before each sampling, the auger was disinfected with alcohol and burned with a butane torch. Subsequently, the four subsamples from each plant were mixed into a single sample (approximately 1,000 g) and kept in a sterile plastic bag to be transported to the laboratory. In this way, a total of 84 samples (6 plants × 14 farms) were obtained (42 from rhizospheric soil of plants with symptoms and 42 from asymptomatic plants). At the same time, vascular tissue samples were extracted from the inner part of the rhizome and the pseudostem of each plant to verify the presence or absence of *Foc*-STR4 in plants with and without symptoms, respectively. In this case, the samples (500–1,000 g/plant) were extracted with a sterile scalpel knife and wrapped in absorbent filter paper. Both types of samples (soil and plant) were transported refrigerated to the laboratory.

### Determination of soil chemical properties

Five hundred grams of each soil sample were processed. The soils were air-dried at room temperature, sieved through a 2 mm sieve and homogenized. Oxidizable organic matter (%) was analyzed by oxidation with potassium dichromate in an acid medium and titrated with Mohr’s salt, using a protocol modified from Walkley and Black (1934). Total nitrogen (%) content was determined by the Kjeldahl method. Assimilable phosphorus (mg/kg) was analyzed according to the colorimetric method of Olsen et al. (1954). Soil exchange cations (mEq/kg), calcium, magnesium, potassium and sodium were extracted with neutral 1N ammonium acetate and determined by flame atomic absorption spectrophotometry (Bower et al., 1952). The pH and the electrical conductivity (EC) were determined in a 1:5 (v:v) aqueous extract according to Norma UNE-EN 77308:2001 (2001) and Norma UNE-ISO 10390:2012 (2012), respectively. The analyses of these parameters were carried out by the Laboratory Unit of the Canary Institute of Agricultural Research (ICIA).

### Microbiological analysis

#### Isolation and preservation of *Trichoderma* isolates from soil samples

Each soil sample was air-dried and sieved (3 mm) to separate the soil conglomerates and larger organic matter remains. Subsequently, 20 g of soil were mixed with 180 ml of sterile 0.85% KCl (potassium chloride, PanReac AppliChem, Barcelona, Spain) in 250 ml capacity bottles and homogenized using an orbital shaker (Orbital, J.P. Selecta) at 150 rpm during 20 min. A serial of decimal dilutions was prepared and 0.1 ml of each dilution was streaked onto the following culture mediums for *Trichoderma* isolation: *Trichoderma* Selective Medium (TSM) (Askew and Laing, 1993) and Dichloran Rose Bengal Chloramphenicol Agar (DRBC, Condalab, Laboratory Conda S.A., Madrid, Spain).

Petri dishes were incubated at 25°C for 7–10 days in the dark. Putative *Trichoderma* colonies were quantified and purified by subculturing on Blakeeslee’s Malt Extract Agar (MEAbl; Blakeslee, 1915; Visagie et al., 2014) for 7 days at 25°C. All isolates described in this study were maintained at −80°C in 30% glycerol solution.

Species were identified by a combination of morphological analysis and molecular methods. Morphological characteristics were based on the key of Gams and Bissett (1998). Colony characteristics were also examined on cultures grown on MEAbl after 7 days of incubation at 25°C. Microscopic observations were made on cultures grown on Corn Meal Agar (CMD; CM0103, Oxoid Ltd., Basingstoke, Hampshire, United Kingdom) and on Spezieller Nährstoffarmer Agar (SNA) after 10 days at 25°C. Observations were made with a Nikon Eclipse 80i optical microscope using differential interference contrast (DIC).

#### Soil and plant pathogen analysis

The quantification of *Foc* was carried out by planting (in triplicate) a serial dilution of the rhizosphere soil suspensions onto Petri dishes containing the Komada selective medium (Komada, 1975). After planting, the Petri dishes were incubated at 25°C for 7–10 days in the dark, and the colonies with microscopic characteristics of *Fusarium* were counted to obtain the number of colony forming units per gram of soil (cfu/g) (Leslie and Summerell, 2006).

The analysis of the plant tissue samples was carried out using the following procedure: four pieces of each type of tissue (corm and pseudostem from each plant) were obtained and surface disinfected. From each of them, a 15–20 mm long portion of the vascular bundles was extracted aseptically and deposited on the surface of a Petri dish with Potato Dextrose Agar (PDA, Condalab, Laboratory Conda S.A., Madrid, Spain), supplemented with streptomycin (300 mg/L) and chloramphenicol (250 mg/L). After incubation at 25°C for 7 days in the dark, putative *Fusarium* colonies were purified by subculturing on PDA. Subsequently, macroscopic (colony diameter, surface appearance, colony edge, coloring, pigmentation in the medium, exudates formation, etc.) and microscopic (shape and arrangement of micro- and macroconidia, length of conidiophores, chlamydospore formation, etc.) characteristics of each isolate were recorded (Leslie and Summerell, 2006) and stored at −80°C in 30% glycerol solution.

### DNA extraction and PCR amplification

DNA from all *Trichoderma* and *Fusarium* isolates was obtained according to the following protocol. Each isolate was grown in PDA for 7–10 days at 25°C. Subsequently, the mycelium was collected and transferred to a microtube (approximately 50–70 mg) with 500 μl of lysis buffer (400 mM Tris–HCl, 60 mM EDTA, 150 mM NaCl, 1% SDS) containing glass beads. The mixture was shaken in a Retsch MM400 shaker (Retsch, Düsseldorf, Germany) for 10 min, incubated at 65°C for 40 min and centrifuged at 15,000 rcf for 10 min. The supernatant was recovered in a sterile microtube and an equal volume of chloroform/isoamyl alcohol [24:1 (v/v)] was added. After homogenization of the mixture and centrifugation at 17,000 rcf (4°C for 10 min), the supernatant was recovered in a sterile microtube. The DNA was precipitated with 2.5 volumes of cold absolute ethanol at −20°C for 2–4 h and collected by centrifugation at 20,000 rcf (4°C for 15 min). The pellet was washed with 500 μl of 70% ethanol, air-dried and resuspended in 50 μl of sterile DNase/RNase-free water. The DNA was quantified using a spectrophotometer (NanoDrop 2000c, Wilmington, NC, USA), and stored at −20°C until use.

For molecular identification of *Trichoderma* species, a 1,200 bp fragment of the translation elongation factor 1-α region (*tef1-α*) gene was amplified by conventional PCR using the following primers pairs: EF1-728F (5′-CAT CGA GAA GTT CGA GAA GG-3′) (Carbone and Kohn, 1999) and TEF1LLErev (5′-AAC TTG CAG GCA ATG TGG-3′) (Jaklitsch et al., 2005). PCR reactions were performed in a volume of 25 μl at the following final concentrations: 1.6 ng/μl of DNA, 1× buffer [Tris–HCl pH 8.5, (NH_4_)_2_SO_4_, and 1% Tween 20], 1.5 mM MgCl_2_, 0.15 mM dNTP, 0.3 μM of each primer, 0.05 U/μl Taq DNA Polymerase (VWR Taq DNA Polymerase). Amplification was carried out in a conventional Thermal Cycler (Eppendorf Mastercycler X50s) under the following conditions: initial denaturation of 5 min at 94°C; 30 cycles of 45 s at 95°C, 45 s at 57°C, 1:10 min at 72°C; with a final extension of 10 min at 72°C.

The PCR product was electrophoresed at 90 V⋅cm^–1^ for about 2.5 h on agarose gels (1.5% w/v) with 1 × TAE buffer (PanReac AppliChem) and stained with gel red (Gel Red Nucleic Acid Gel Stain, Biotium). A 50-bp DNA Step Ladder (S7025, Sigma-Aldrich) was used as a size standard. The PCR products were visualized under UV light and photographed (Nikon D3500 DX, Nikon).

For the molecular identification of the *Foc* isolates obtained from the plant tissue samples (rhizome and pseudostem), a PCR amplification was performed using secreted in xylem (SIX) specific primers to detect the different *Foc* races: STR4: *Foc*-SIX8b-F/*Foc*-SIX8b-R (Fraser-Smith et al., 2014) and SIX8b-206-F/SIX8b-206-R (Carvalhais et al., 2019); TR4: SIX1a-266-F/SIX1a-266-R (Carvalhais et al., 2019). Subsequently, all the isolates with positive results in the specific amplifications were confirmed and identified by partial sequencing of the translation elongation factor 1-α gene (*tef1-α*) using the primers EF-1 and EF-2 (O’Donnell et al., 1998). The PCR conditions used for each primer set were those recommended by the respective authors. The fragments were evaluated by electrophoresis in agarose gel as described above.

### Sequence and phylogenetic analysis

The PCR products were purified with exonuclease I (M0293S, BioLabs) and shrimp alkaline phosphatase (M0371S, BioLabs) according to the manufacturer instructions [Shrimp Alkaline Phosphatase (rSAP), BioLabs]. Purified amplicons were sequenced through Sanger sequencing methods by Macrogen sequencing service (Macrogen Inc., Spain) in both directions (forward and reverse complimentary DNA strands). The sequence data assembly and editing were performed using MEGA 11 software (Tamura et al., 2021).

All the *Trichoderma* sequences were deposited in GenBank database with the accession numbers from OQ858692 to OQ858800 (Supplementary Table 2). Comparisons with sequences from GenBank were performed using BLASTN (Altschul et al., 1997). Moreover, sequences of the *tef1-α* region were compared with those available at the TrichOKEY databases (TrichOKEY v2 software), accessed online at the International Subcommittee on *Trichoderma* and *Hypocrea* taxonomy (ISTH)^2^ (Druzhinina et al., 2005). For phylogenetic analysis, sequences of the representative species of the *Trichoderma* genus were retrieved from the NCBI GenBank database^3^ and are listed in Supplementary Table 3. Multiple sequence (*tef1-α* dataset) alignments were performed with MUSCLE and phylogenetic trees were reconstructed using maximum-likelihood (ML) analysis using MEGA 11 software. The robustness of branches was assessed by bootstrap analysis of 1,000 replicates. For ML analyses, the best-fit nucleotide substitution model for each dataset was selected with MEGA 11, according to the Bayesian information criterion (BIC) values (Schwarz, 1978). In this context, the best models (lowest BIC scores) were Kimura 2-parameter model with a gamma distribution (+G) and invariable sites (+I) for the *Trichoderma* lineage, *Longibrachiatum* lineage and *Harzianum-Virens* lineage.

*Fusarium* sequences (*tef1-α*) were analyzed using a procedure similar to that described for *Trichoderma*. In this case, the best model for the ML analysis of the *F. oxysporum* species complex (FOSC; Lombard et al., 2019) was the Kimura 2-parameter model with a gamma distribution (+G).

### Diversity analysis of *Trichoderma* species

Different indices were analyzed to quantitatively determine the diversity of *Trichoderma* species in the different bioclimatic zones (northern and southern slopes) according to the origin of the samples (soil rhizosphere of plants with and without *Foc*-STR4).

The occurrence frequency (OF) percentage at the species level was calculated using the following formula:


OF(%)=nN 100

where “*n*” is the number of rhizospheric soil samples with one *Trichoderma* species and “*N*” is the total number of rhizospheric soil samples.

The relative abundance (RA) percentage for every species was calculated as:


RA(%)=n⁢iN⁢t 100

where “ni” is the number of isolated of *Trichoderma* belonging to species *i* and “Nt” is the total number of isolates.

The isolation rate (IR) was calculated by the total number of isolates of *Trichoderma* (nt) divided by the total number of soil samples (Ns).


$$I\,R=\frac{n\,t}{N\,s}$$

Margalef’s (E) index was used to measure the richness (Margalef, 1958), Shannon–Wiener (H), and Simpson (D) indices were used to measure the diversity (Shannon, 1948; Simpson, 1949), and Pielou index (J) was used to measure evenness (Pielou, 1966). These indices were calculated using the following formulas:


$$E=\frac{S-1}{\ln N}$$

$$H=-\sum_{i=1}^{N}P_{i}\,l\,n\,P_{i},P_{i}=\frac{n_{i}}{N}$$

$$D=1-\left(\Sigma \,{\text{Pi}}^{2}\right)$$

$$J=\frac{H}{l\,n\,S}$$

where “*S*” represents the number of *Trichoderma* species, “*N*” is the sum of all *Trichoderma* species isolates, “*P*_*i*_” is the ratio of the number of isolates of *Trichoderma* belonging to species *i* (*n*_*i*_) to the total number of isolates in the community (*N*).

### Data processing

Microsoft Excel version 2016 software was used to calculate the diversity indices. Statgraphics Centurion 18 V18.1.14 software was used for statistical analysis of the data. One-way analysis of variance (ANOVA) and LSD (*p* < 0.05) multiple comparisons were used to analyze the variance and test the significance of differences in soil chemical properties and *Foc* population. The hierarchical location of the *Trichoderma* species detected in the rhizospheric soil samples, was determined using the Olmstead-Tukey correlation method. The graphical representation used a quadrant graph based on the occurrence frequency and the relative abundance [expressed as Log (RA + 1)] values (Sokal and Rohlf, 1995). Each species was classified as dominant (OF and RA higher than the average values), frequent (OF above average and RA below average), occasional (OF below average and RA above average), and rare (OF and RA lower than the average values). The Spearman correlation coefficient method was used to analyze the correlation between soil chemical properties and *Trichoderma* community composition.

## Results

### Analysis of *Fusarium oxysporum* f. sp. *cubense* in plant samples and quantification in soil

To test the absence and presence of the pathogen on healthy and *Foc*-STR4-affected banana plants, respectively, rhizome and pseudostem samples were obtained from all banana plants selected for soil sampling. Endophytic fungi were isolated from these samples and all colonies with macro and microscopic characteristics typical of *Fusarium* were identified at species and race level by molecular and phylogenetic analysis of the *tef1-α* gene and specific-race SIX primers (O’Donnell et al., 1998; Fraser-Smith et al., 2014; Carvalhais et al., 2019). The results of this analysis determined that the causal agent of the disease in plants with symptoms was *Foc*-STR4 (*Fusarium phialophorum*), while in plants without symptoms, the pathogen was not detected in any case.

**FIGURE 1:**
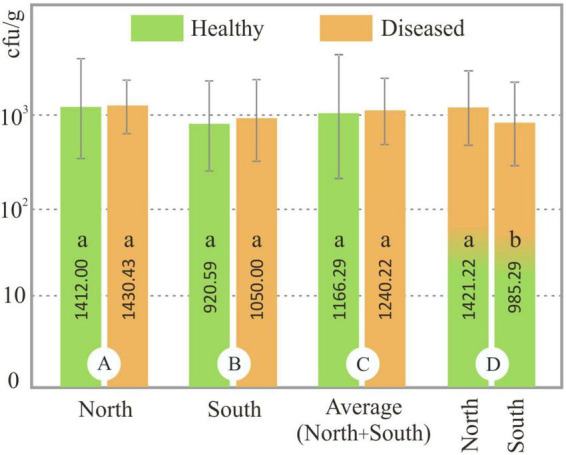
*Fusarium oxysporum* f. sp. *cubense* (*Foc*) population in soil samples. Columns in group A and B show the *Foc* population (cfu/g) in soils from healthy and diseased plants from the northern and southern slopes, respectively. Columns in group C show the *Foc* population of healthy and diseased plants from all soil samples (north + south). Columns of group D show the *Foc* population in all soils (healthy + diseased) of the north and south slope. Equal letters in the same group of data (A, B, C or D) indicate no significant difference (*p* < 0.05). Errors bars represent the standard deviations of the means.

The abundance of *Foc* in the rhizosphere of healthy and diseased plants was analyzed in each soil sample. No significant difference (*p* < 0.05) was observed between the population of *Foc* (ufc/g) of the healthy and diseased soils on both slopes (north and south). However, significant differences (*p* < 0.05) were detected between northern and southern soils samples (Figure 1). Our results showed that population of *Foc* in northern soils ranged from 3.67 × 10^2^ to 3.63 × 10^3^ cfu/g (average 1.41 × 10^3^ cfu/g) and 1.50 × 10^2^ to 2.20 × 10^3^ cfu/g (average 1.43 × 10^3^ cfu/g) in healthy and diseased plants, respectively, and in southern soils ranged from 0.83 × 10^2^ to 1.68 × 10^3^ cfu/g (average 9.21 × 10^2^ cfu/g) and 9.75 × 10^2^ to 1.46 × 10^3^ cfu/g (average 1.05 × 10^3^ cfu/g) in healthy and diseased plants, respectively (Figure 1). The lowest *Foc* population in the rhizosphere of healthy plants was detected in CPE (Cueva del Polvo, southern slope) and the highest was in VO78 (Valle de La Orotava, northern slope). The lowest and highest populations in diseased plants were detected in BN47 and BN4 (Buenavista del Norte, north slope), respectively.

### *Trichoderma* isolation and species identification

The most appropriate culture medium for *Trichoderma* isolation was DRBC. This was mainly due to the following reasons: (a) the development and sporogenesis of *Trichoderma* colonies on DRBC was significantly higher than on TSM, (b) the contrasting color of the rose bengal of DRBC facilitated the recognition of *Trichoderma* colonies, and (c) most of the colonies corresponding to the soil mycobiota population did not show abundant growth. Therefore, the *Trichoderma* colonies in DRBC were quickly located and identified by micromorphological characteristics.

No significant differences (*p* < 0.05) in *Trichoderma* population were observed between healthy and diseased soils, and between northern and southern soils. Nevertheless, *Trichoderma* quantification (cfu/g) on samples from healthy plants was higher than in diseased plants (3.4 × 10^3^ and 2.1 × 10^3^ cfu/g, respectively). Likewise, the cfu/g from soils on the northern slope were slightly higher than those from soils on the southern slope (2.9 × 10^3^ and 2.7 × 10^3^ cfu/g, respectively). Regarding the relationship between *Trichoderma* and *Fusarium* populations in soil, no significant correlation was observed between both populations in any of the conditions tested (north and south slope or healthy and diseased plants).

**FIGURE 2:**
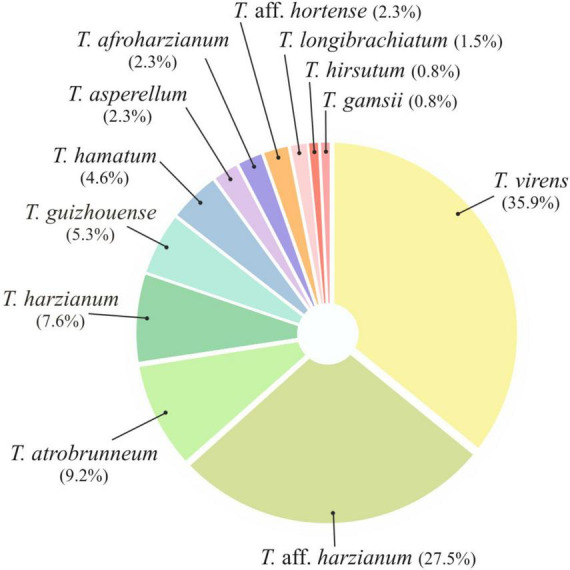
Pie chart showing the relative abundance (%) of each *Trichoderma* species isolated in this study from banana rhizosphere soil in Tenerife (Canary Islands).

Taking into account the morphological (macro and microscopic) diversity of the colonies and the origin of the samples, a total of 131 *Trichoderma* isolates were obtained from 84 soil samples collected from 14 farms located in different banana-production areas of Tenerife (8 farms from the north slope and 6 from the south). All *Trichoderma* isolates were identified at species level by the sequence analysis of *tef1-α* gene. Twelve species were identified: *T. virens* (47 isolates), *T.* aff. *harzianum* (36), *T. atrobrunneum* (12), *T. harzianum* (10), *T. guizhouense* (7), *T. hamatum* (6), *T.* aff. *hortense* (3), *T. afroharzianum* (3), *T. asperellum* (3), *T. longibrachiatum* (2), *T. gamsii* (1), and *T. hirsutum* (1) (Figure 2).

### Phylogenetic analysis

One hundred nine isolates were selected for taxonomy studies according to the macro and micromorphological characteristics of the 131 isolates initially obtained from the soil samples of banana plants in Tenerife. The phylogenetic relationship of the 109 representative isolates of *Trichoderma* was constructed from the sequence analysis of *tef1-α* gene using the ML method. The 12 identified species were distributed among three different evolutionary lineages of the *Trichoderma* genus namely: *Harzianum-Virens* (8 species, 90.84% of the isolates), *Trichoderma* (3 species, 7.63% of the isolates), and *Longibrachiatum* (1 species, 1.53% of the isolates) (Cai and Druzhinina, 2021; Gutiérrez et al., 2021).

**FIGURE 3:**
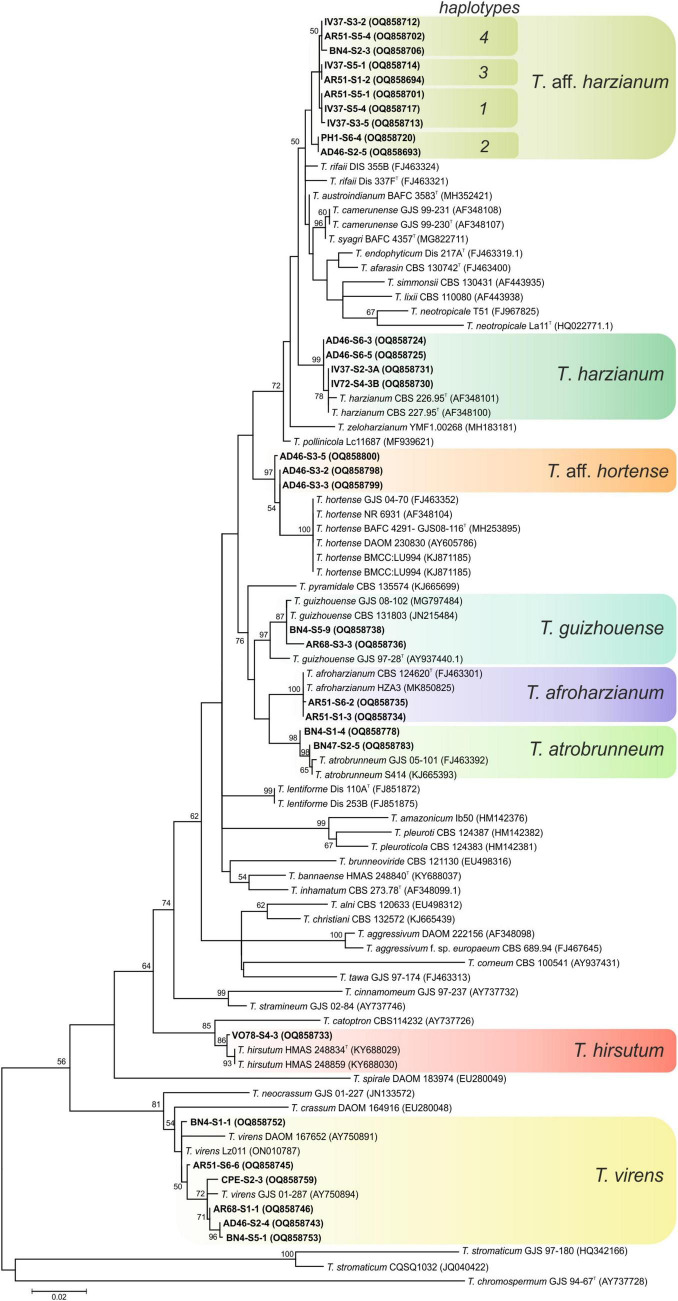
Maximum likelihood phylogenetic tree of the *Harzianum-Virens* lineage (Jaklitsch and Voglmayr, 2015) showing the position of seven *Trichoderma* species isolated from banana rhizosphere soils in Tenerife (Canary Islands) based on the nucleotide sequences of the *tef1-α* gene. Sequences obtained in this work are indicated in bold. ML bootstrap support above 50% is given on each node. The type strains are indicated with “^T^” . GenBank accession numbers are shown in parentheses.

**FIGURE 4:**
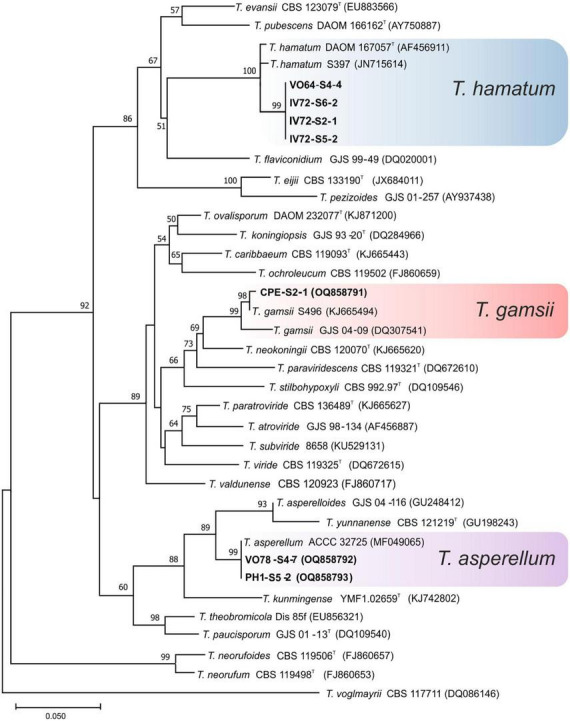
Maximum likelihood phylogenetic tree of the *Trichoderma* lineage (Jaklitsch and Voglmayr, 2015) showing the position of three *Trichoderma* species isolated from banana rhizosphere soils in Tenerife (Canary Islands) based on the nucleotide sequences of the *tef1-α* gene. Sequences obtained in this work are indicated in bold. ML bootstrap support above 50% is given on each node. The type strains are indicated with “^T^” . GenBank accession numbers are shown in parentheses.

**FIGURE 5:**
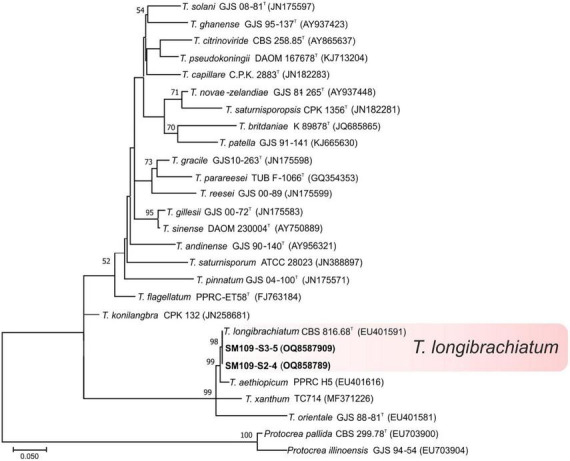
Maximum likelihood phylogenetic tree of the *Longibrachiatum* lineage (Jaklitsch and Voglmayr, 2015) showing the position of one *Trichoderma* species isolated from banana rhizosphere soils in Tenerife (Canary Islands) based on the nucleotide sequences of the *tef1-α* gene. Sequences obtained in this work are indicated in bold. ML bootstrap support above 50% is given on each node. The type strains are indicated with “^T^” . GenBank accession numbers are shown in parentheses.

Ninety-seven isolates were identified into six known species belonging to the linage *Harzianum-Virens* (Jaklitsch and Voglmayr, 2015); *T. afroharzianum, T. atrobrunneum*, *T. guizhouense, T. harzianum*, *T. hirsutum*, and *T. virens* (Figure 3). Ten isolates were identified in the *Trichoderma* linage (Jaklitsch and Voglmayr, 2015) as *T. asperellum, T. gamsii*, and *T. hamatum* (Figure 4), and two isolates were identified in the *Longibrachiatum* linage as *T. longibrachiatum* (Jaklitsch and Voglmayr, 2015; Figure 5).

Interestingly, 34 isolates could not be grouped in previously described *Trichoderma* species. In these cases, the term *affinis* (aff.) was used to indicate that these OTUs are similar but not necessarily identical to the described species. Thus, 31 isolates were named *T.* aff. *harzianum* and 3 isolates as *T.* aff. *hortense*, both belonging to the *Harzianum-Virens* lineage. The phylogram showed that *T.* aff. *harzianum* formed a subclade with *T. afarasin, T. austroindianum, T. camerunense, T. endophyticum, T. lixii, T. neotropicale, T. rifaii, T. simmonsii*, and *T. syagri*, and *T.* aff. *hortense* formed a subclade with *T. hortense. Trichoderma* aff. *harzianum tef1-α* sequence allowed the detection of four haplotypes (haplotype 1: 13 isolates; haplotype 2: 9 isolates; haplotype 3: 5 isolates; and haplotype 4: 4 isolates). The comparison with sequences of the type strains of the related species showed 98.2% similarity (4 substitutions + 2 gaps) with *T. austroindianum* VAB T050*^T^* (accession no. MH352421), 97.2% similarity (9 substitutions + 1 gaps) with *T. syagri* BAFC 4357*^T^* (MG822711), 97.0% similarity (10 substitutions + 1 gaps) with *T. camerunense* GJS99 230*^T^* (AF348107), 96.1% similarity (8 substitutions + 7 gaps) with *T. rifaii* DIS 37F*^T^* (FJ463321), and 94.46% similarity (12 substitutions + 3 gaps) with *T. harzianum* CBS 226.95*^T^* (AF348101). In addition, *T.* aff. *hortense* showed 95.6% similarity (4 substitutions + 10 gaps) with the type strain of *T. hortense* G.J.S. 08-116*^T^* (accession no. MH253895).

### Diversity and distribution of *Trichoderma* species in the rhizosphere of healthy and *Foc*-STR4-affected banana plants in different agroecosystems

*Trichoderma* isolates were obtained from all the farms analyzed and from 65.48% of soil samples examined (70.83% in the soils of the northern slope and 58.33% in soils of the southern slope). In relation to the number of isolates (131 in total), 75 isolates were obtained from the soils of the northern slope (57.25% of the total isolates) and 56 from the soils of the south of the island (42.75% of the total isolates) (Table 2). In general terms, it can be observed that the percentage of soil samples with *Trichoderma* and the percentage of isolates obtained is higher on the northern slope than on the southern slope. In addition, on both slopes, the percentage of isolates obtained from healthy plants is higher than that of diseased plants (51.91% and 48.09%, respectively).

**TABLE 2:** *Trichoderma* isolates’ distribution in the different banana rhizosphere soils in Tenerife.

	Northern slope		Southern slope	
	Diseased plants	Healthy plants	Diseased plants	Healthy plants
Number of farms	8		6	
Number of soil samples	24	24	18	18
Percentage of soil samples with *Trichoderma* isolates	70.83		58.33	
	62.50	79.17	55.56	61.11
Number of *Trichoderma* species	7	8	8	8
Number of *Trichoderma* isolates	75		56	
	36	39	27	29
Isolation rates (IR)	1.50	1.62	1.50	1.61
Percentage of isolates	57.25^(1)^		42.75^(1)^	
	27.48^(1)^	29.77^(1)^	20.61^(1)^	22.14^(1)^
	48.00^(2)^	52.00^(2)^	48.21^(3)^	51.79^(3)^

^(1)^In relation to the total number of isolates (131). ^(2)^In relation to the number of isolates from northern soils (75). ^(3)^In relation to the number of isolates from southern soils (56).

**FIGURE 6:**
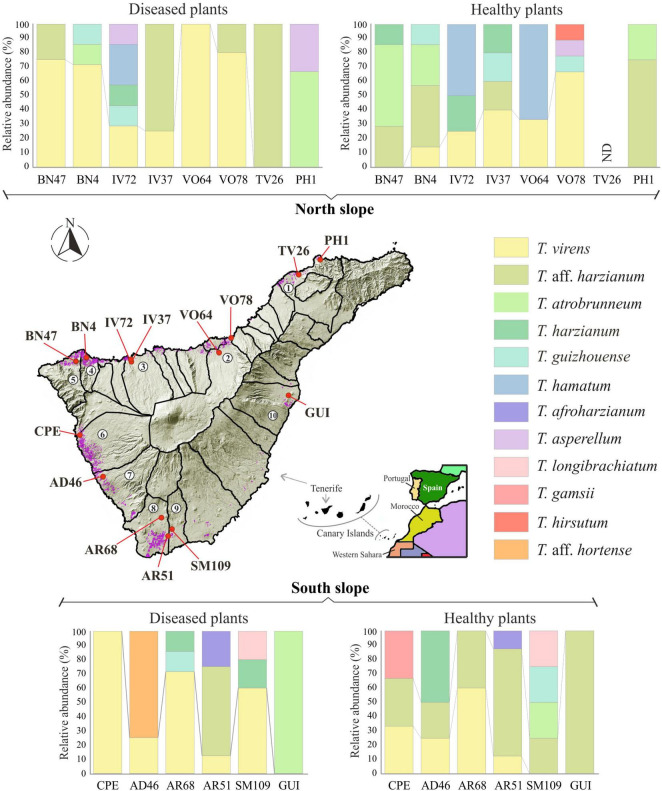
Diversity and distribution of *Trichoderma* species in banana rhizosphere soil in Tenerife (Canary Islands). Sampling points and farm locations are indicated with a red circle. Banana growing areas are indicated in purple. Municipalities are indicated with numbers: 1, San Cristóbal de La Laguna; 2, Valle de La Orotava; 3, Icod de los Vinos; 4, Los Silos; 5, Buenavista del Norte; 6, Guía de Isora; 7, Adeje; 8, Arona; 9, San Miguel de Abona; 10, Güímar; ND: *Trichoderma* was not detected.

**FIGURE 7:**
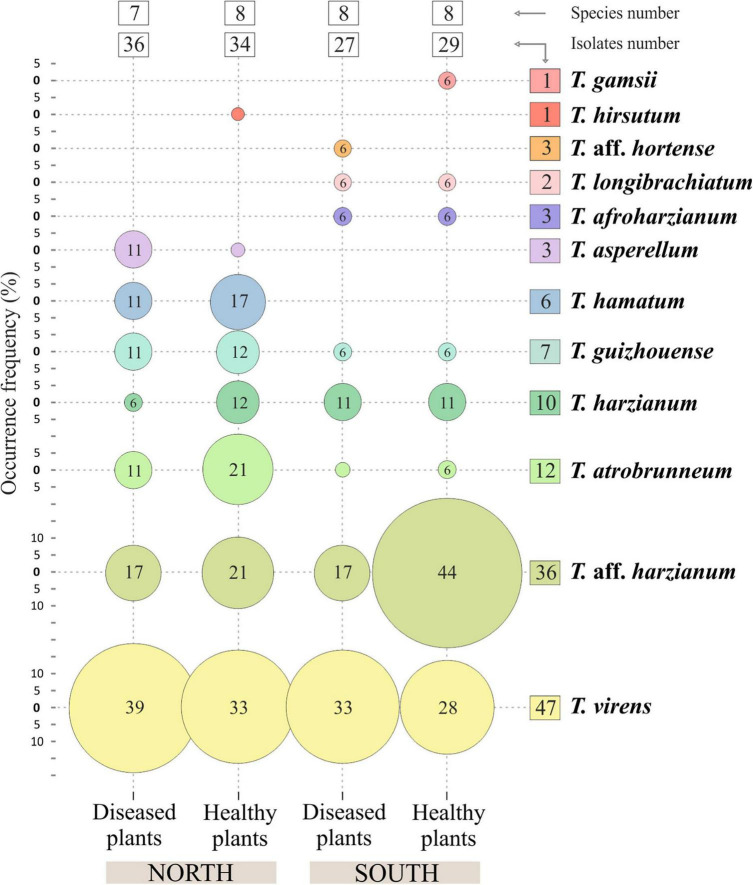
Occurrence frequency (OF), number of isolates and species of *Trichoderma* spp. in banana rhizosphere soils in Tenerife (Canary Islands). OF above 5% are indicated inside the circles.

Regarding the number of species identified (12 in total), no differences were observed between the different origins of the samples. In the northern soils, a total of eight species were identified, while from the southern soils, nine species were identified. *Trichoderma virens* and *T. harzianum* group (identified as *T.* aff. *harzianum* and *T. harzianum*) were the most abundant species. *Trichoderma virens* represent 35.9% of isolates and was detected in 11 of the 14 farms (78.6% of the farms). *Trichoderma* aff. *harzianum* and *T. harzianum* represent 35.1% of all isolates and were detected in 12 and 6 farms, respectively (85.7% and 42.8% of the farms). It is interesting to note that *T.* aff. *harzianum* was the only species isolated in all farms on the southern slope from the soil of healthy plants (Figure 6). *Trichoderma atrobrunneum* (9.2% of isolates) and *T. guizhouense* (5.3%) were the most abundant species after *T. virens* and *T.* aff. *harzianum*, being observed in 5 and 6 farms, respectively. *Trichoderma hamatum* and *T. asperellum* (4.6% and 2.3% of isolates, respectively) were detected on two farms and *T. hirsutum* (0.76%) on one farm on the northern slope, while none of these species were detected on the southern slope (Figures 6, 7). Unlike *T. afroharzianum* (2.3% of isolated), *T.* aff. *hortense* (2.3%), *T. longibrachiatum* (1.5%), and *T. gamsii* (0.76%) were detected in one farm on the southern slope, while no isolates were obtained from northern slope (Figures 6, 7).

**FIGURE 8:**
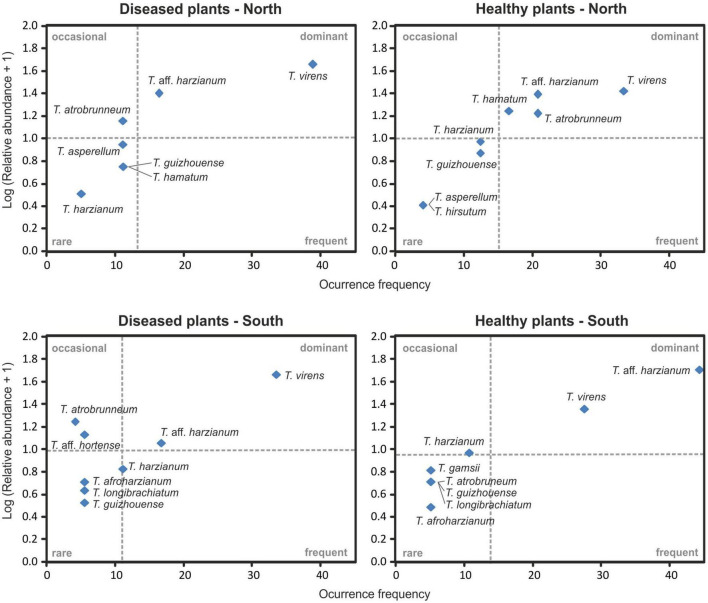
Olmstead-Tukey diagram relationship between the occurrence frequency and the relative abundance of *Trichoderma* species identified from banana rhizosphere soil on the north and the south slopes of Tenerife. The dashed lines correspond to the mean relative abundance (horizontal) and occurrence frequency (vertical) and are used to define the dominant, frequent, occasional, and rare species.

The Olmstead-Tukey scattergram shows *T. virens* and *T.* aff. *harzianum* are the dominant species in the northern and southern soils of healthy and *Foc*-STR4-affected banana plants (Figure 8). Similarly, *T. atrobrunneum* and *T. hamatum* occur as dominant species in the northern soils of healthy plants, while the rest of the species were classified as occasional or rare. The Margalef (E), Shannon (H), Simpson (D), and Pielou (J) diversity indices show a different performance of the species on each slope (Table 3). The richness index (Margalef) of the northern slope was higher in healthy plants (E: 1.91) than in diseased plants (E: 1.82). While on the southern slope, this tendency was not observed. The Simpson index was close to 1, indicating a high diversity of *Trichoderma* species in the banana rhizosphere soils of Tenerife. In northern soils, the diversity of *Trichoderma* of the healthy plants (D: 0.81) were higher than those from diseased plants (D: 0.69). Likewise, the Shannon index showed the same trend (H: 1.82 in healthy plants and H: 1.53 in diseased plants). However, on the southern slope, the diversity indices of healthy plants were lower than those of diseased plants. Pielou’s index measures evenness. It can vary between 0 and 1, where 0 means that there is only one species (no evenness) and 1 means that all species are equally abundant (complete evenness). In this study, we observed that northern soils showed higher species evenness in healthy plants (J: 0.87) than in diseased plants (J: 0.78). However, the trend in the southern soils is the opposite: soils of healthy plants (J: 0.75) have a lower index than soils of diseased plants (J: 0.80). With regard to the relationship between soil *Foc* population (cfu/g) and the *Trichoderma* biodiversity indices, no significant correlation was detected between both parameters.

**TABLE 3:** Diversity indices of *Trichoderma* species isolated from healthy and diseased banana plants of the northern and southern slope of the island of Tenerife.

Slope	Plants	Margalef (E)	Simpson (D)	Shannon (H)	Pielou (J)
North	Healthy	1.91	0.81	1.82	0.87
	Diseased	1.82	0.69	1.53	0.78
South	Healthy	2.08	0.72	1.56	0.75
	Diseased	2.12	0.74	1.67	0.80

### Effect of soil chemical properties on *Trichoderma* community

To evaluate the effect of the soil chemical properties on the relative frequency of the different *Trichoderma* species, the oxidizable organic matter, total nitrogen, assimilable phosphorus, calcium, magnesium, potassium, sodium, electrical conductivity, and pH were determined in the soil samples of each of the farms. No significant differences (*p* < 0.05) were observed (on both slopes) between the chemical properties of healthy and diseased soils. However, the comparison of the soils from the northern and southern slopes showed significant differences (*p* < 0.05) in the content of oxidizable organic matter (%), sodium (mEq/kg) and pH (Table 4).

**TABLE 4:** Chemical properties of the different rhizosphere soil samples (healthy and diseased banana plants) obtained from farms located on the northern and southern slopes of Tenerife.

Slope	Plants	Organic matter (%)	Total nitrogen (%)	Phosphorus (mg/kg)	Calcium (mEq/Kg)	Magnesium (mEq/Kg)	Sodium (mEq/Kg)	Potassium (mEq/Kg)	pH	Conductivity (mS/cm)
North	Diseased	5.72 a	0.256 a	36.00 a	232.50 a	134.01 a	34.16 a	42.75 a	7.43 a	0.68 a
	Healthy	5.44 a	0.255 a	34.75 a	241.88 a	123.88 a	33.81 a	36.70 a	7.40 a	0.65 a
	Average	5.58 A	0.256 A	35.38 A	237.19 A	128.94 A	33.99 A	39.73 A	7.41 A	0.67 A
South	Diseased	3.53 a	0.208 a	28.25 a	233.50 a	143.23 a	56.23 a	42.68 a	8.08 a	1.72 a
	Healthy	3.56 a	0.207 a	30.42 a	230.50 a	147.22 a	54.28 a	34.48 a	8.00 a	1.68 a
	Average	3.55 B	0.208 A	29.33 A	232.00 A	145.23 A	55.26 B	38.58 A	8.04 B	1.70 A

Different letters in each column indicate that there are statistically significant differences (Tukey HSD test, *p* < 0.05) between soils from healthy and diseased plants from the same slope (north or south) (lowercase) or between soils from different slopes (uppercase). Each experiment was repeated three times. See section “Materials and methods” for details on chemical analysis of soil samples.

**FIGURE 9:**
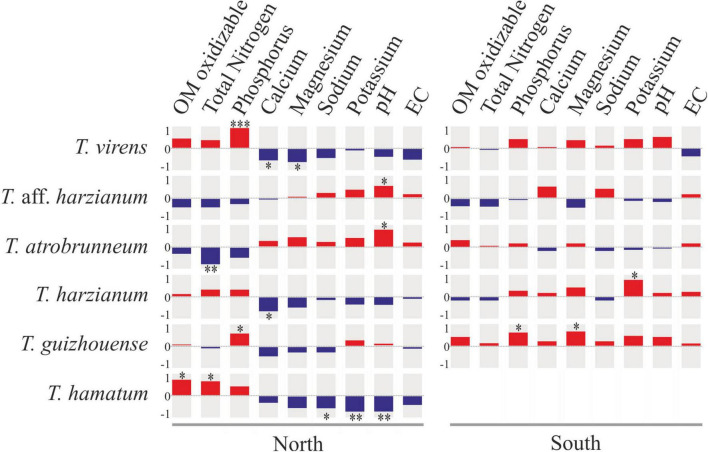
Spearman’s rank correlation matrix between the occurrence frequency of the top six *Trichoderma* species and soil chemical parameters on the northern and southern slopes of Tenerife. Square size reflects the magnitude of the correlation coefficient (red, positive correlation; blue, negative correlation). *p < 0.05; **p < 0.01; ***p < 0.001.

The Spearman analysis showed that certain *Trichoderma* species present significant correlations with some soil chemical parameters. For example, on the northern slope, *T. virens* (the species isolated with the highest occurrence frequency) showed a significant positive correlation with phosphorus content and a negative correlation with calcium and magnesium content, while on the southern slope these correlations were not significant (Figure 9). *Trichoderma* aff. *harzianum* (another species isolated with high occurrence frequency) and *T. atrobrunneum* showed a significant positive correlation with soil pH on the northern slope, while on the southern slope none of the correlations were significant. Likewise, *T. atrobrunneum* showed a significant negative correlation with total nitrogen on the northern slope. *Trichoderma harzianum* showed a significant positive correlation with the potassium content on the southern slope and a negative correlation with calcium on the northern slope. *Trichoderma guizhouense* showed a significant positive correlation with the phosphorous content on both slopes and with magnesium on the southern slope of the island. *Trichoderma hamatum* showed a positive correlation with oxidizable organic matter and total nitrogen and a negative correlation with sodium, potassium, and pH. According to these results, the parameters that significantly affect the *Trichoderma* population were the phosphorus and soil pH.

## Discussion

It is widely recognized that soil features, plant characteristics, microorganism diversity, agronomic management, and environmental factors significantly influence the plant/root-microorganism relationship (Lemanceau et al., 1995). Likewise, the amount of phytopathogen in the soil and its relationship with BCA microorganisms is another important aspect to consider. Xue et al. (2015) found significantly lower colony forming unit numbers of *F. oxysporum* f. sp. *cubense* in the disease-suppressive rhizospheric soil (no disease plants) (approximately 5 × 10^3^ cfu/g) compared to the wilted banana rhizosphere (disease plants) (approximately 1 × 10^5^ cfu/g). Likewise, Zhou et al. (2019) quantified a *F. oxysporum* f. sp. *cubense* population less than 1.3 × 10^3^ cfu/g in disease-free soils and more than 1.7 × 10^3^ cfu/g in diseased soil samples of three banana farms in China. Based on these results, the authors indicate that a *Foc* population lower than 1.3 × 10^3^ cfu/g does not cause *Fusarium* wilt banana in the field. However, these data are not in agreement with our results where 41.6% of diseased soil samples had less than 1.3 × 10^3^ cfu/g, and no significant differences were detected between the rhizosphere *Fusarium* population of healthy and diseased plants (Figure 1). On the other hand, soil chemical parameters can play an important role in the amount of the pathogen. In this regard, Senechkin et al. (2014) and Zhou et al. (2019) describe a positive correlation between soil organic matter and *Fusarium* abundance, and a negative correlation for pH. In this aspect, the results of our work are consistent with these authors: the *Foc* population in the soils of the northern slope was significantly higher than in the southern soils, where organic matter and pH were higher and lower, respectively (Figure 1 and Table 4).

*Trichoderma* is a fungus with a worldwide distribution and widely studied in different natural and agricultural ecosystems of the world. However, there are few works related to *Trichoderma* distribution and biodiversity in banana rhizosphere soils under different environmental conditions or in relation to the health status of the plant. In the specific case of the Canary Islands, Zachow et al. (2009) analyzed the fungal diversity in the rhizosphere of endemic plants of six different climatic and vegetation zones in Tenerife, indicating that it is an interesting place to study biodiversity and species diversification. However, in contrast to other fungi analyzed, the *Trichoderma* species described were ubiquitous and widely distributed and no significant differences were detected between the biodiversity of the different sampling areas. Moreover, Jaklitsch and Voglmayr (2015) analyzed *Trichoderma* populations in Tenerife, La Palma, and La Gomera (the western Canary Islands) and identified 17 described species and 3 putatively new *Trichoderma* species, of which 62% belonged to the Viride clade and 16.7% to *Trichoderma harzianum* s.l.

*Trichoderma harzianum* is a cosmopolitan species widely distributed in different parts of the world, such as Tunisia (Sadfi-Zouaoui et al., 2009), Sardinia (Migheli et al., 2009), Egypt (Gherbawy et al., 2004), South America (Barrera et al., 2021), Canary Islands (Tenerife) (Zachow et al., 2009), etc. Moreover, it is the species with the highest number of citations in soil samples from banana crops: China (Yang et al., 2016; Du et al., 2020), India (Thangavelu et al., 2004; Thangavelu and Gopi, 2015), Malaysia (Naher et al., 2019), Mexico (Hernández-Domínguez et al., 2019), and Tenerife (Canary Islands) (Ciancio et al., 2022). In our study, this species was detected in healthy and *Foc*-STR4 diseased plants on both slopes of the island (Figures 6–8). Likewise, *T.* aff. *harzianum* (a putatively new species) was one of the dominant species (27.48% of the isolates) (Figures 2, 8) and was detected on both slopes of the island in healthy and diseased plants (Figure 7). Notably, it was the only species detected in 100% of the southern farms in the rhizosphere of healthy plants (Figure 6). Additionally, a positive correlation (*p* < 0.05) with the soil pH was detected on the northern slope (Figure 9). These results agree with those described by Mayo-Prieto et al. (2021), who observed an increase in the development of *T. harzianum* T059 in Esla-Campo’s soils with high pH values.

*Trichoderma virens* was the most frequently isolated species (35.89% RA) (Figure 2). On the northern slope, it was detected in 62.5% and 75.0% of the farms (from healthy and diseased soils samples, respectively), while on the southern slope it was observed in 66.7% and 83.3% of the farms (healthy and diseased soils, respectively) (Figures 6, 7). According to the Olmstead-Tukey test, *T. virens* was classified as a dominant species on both slopes of the island (Figure 8) and in the north, its abundance was significantly positively correlated (*p* < 0.001) with the phosphorus content in the soil (Figure 9). Surprisingly, this species has not been previously described in banana soils elsewhere in the world and, in Tenerife, it was identified by Ciancio et al. (2022) from a “control” sample of rhizospheric soil free of banana roots. *Trichoderma virens* is a potent bioeffector for plant protection (biofungicide) and plant growth promotion (biofertilizers) (Harman et al., 2004) and is currently marketed as a BCA in organic farming (Woo et al., 2023). Muthukathan et al. (2020) identified *T. virens* proteins involved in banana/root-*Trichoderma* interaction that could be relevant for disease management in banana. These proteins are associated with penetration, colonization and induction of the plant defense response. For this reason, finding this dominant species in the banana soils rhizosphere in Tenerife opens a promising avenue of research into the selection of potential *Foc*-STR4 biocontrol agents adapted to the environmental and crop conditions of the Canary Islands.

*Trichoderma atrobrunneum* is a widely distributed species and has been described in different parts of the world in soil samples or decaying wood of North America, Europe (Jaklitsch and Voglmayr, 2015), and Africa (Haouhach et al., 2020; Nyang’au et al., 2023). However, none of the previous works performed in the Canary Islands or in banana soils in other parts of the world describe this species. In our work, the highest occurrence frequency in the rhizosphere of healthy plants from the northern slope was observed (Figures 6–8) and a significant positive correlation (*p* < 0.05) with the soil pH of the northern slope was detected (Figure 9). Therefore, this is the first time it has been described in Tenerife and in banana rhizosphere.

The ability of *T. atrobrunneum* as BCA has been demonstrated in many studies. The genomic analysis reveals the presence of different genes encoding for carbohydrate-active enzymes, proteins associated with the synthesis of secondary metabolites, peptaboils, epidithiodioxopiperazines, and siderophores potentially involved in parasitism, saprophytic degradation as well as in biocontrol and antagonistic activities (Fanelli et al., 2018). Pavlovskaya et al. (2020) demonstrate the ability to control *Fusarium* wilt in cucumber through an efficient protective effect and growth stimulation. Natsiopoulos et al. (2022) report the efficacy of an isolate obtained in Serres (Northern Greece) from corn cobs to significantly reduce the *in vitro* development of *F. oxysporum* f. sp. *lycopersici* and to decrease the incidence of the disease in tomato plants grown in pots. This background demonstrates the biocontrol capacity of *T. atrobrunneum* and its potential use in the control of *Foc*-STR4 on banana crops in the Canary Islands.

*Trichoderma guizhouense* was first described in soil samples from two different regions in Guizhou province in China (Li et al., 2013) and has subsequently been detected in different countries in southern Europe (Croatia, Italy, Greece, and Spain) and South America (Argentina) (Jaklitsch and Voglmayr, 2015; Barrera et al., 2021). However, in the Canary Islands, previous works do not describe this species, whereas in our study, we obtained isolates from both slopes of the island in the rhizosphere of healthy and diseased plants (Figures 6–8). Furthermore, a significant positive correlation (*p* < 0.05) was detected with the phosphorus content of the soil on both slopes (Figure 9). Regarding its agronomic utility, several studies have demonstrated the ability of this species to promote plant growth, produce volatile compounds and antioxidants, and control the development of phytopathogens (Wang and Zhuang, 2019; Liu et al., 2021). In this regard, the NJAU 4,742 strain isolated in China from aromatic plant tissue has been intensively studied for its antifungal capacity against different plant pathogens of different crops, including *Foc*-TR4 (Zhang et al., 2016, 2019; Pang et al., 2020).

*Trichoderma hamatum* has been described in banana soils in Mexico with a relative abundance of 5% (referring to the total number of *Fusarium* and *Trichoderma* isolates evaluated) (Hernández-Domínguez et al., 2019). In our work, this species constituted 4.6% (RA) of the *Trichoderma* isolates (Figure 2) and was only detected in samples from the northern slope as a dominant species in healthy plants and rare in diseased plants (Figures 7, 8). Previous works in the Canary Islands have not reported this species, while in other parts of the world it is described as a widely distributed species in different environmental conditions. Danielson and Davey (1973) and Jiang et al. (2016) highlight the ability of *T. hamatum* to tolerate excessive moisture conditions in forest soils in the United States (North Carolina, Virginia, and Washington) and in farmland soils of East China, respectively. However, Sadfi-Zouaoui et al. (2009) found *T. hamatum* to be a dominant species in all areas of Tunisia and detected no differences in its distribution with respect to different climatic conditions. By contrast, in Tenerife, the distribution of this species seems to be affected by the climatic and soil conditions of the slope, with the northern area being more favorable for its development (Figures 6–8). Additionally, highlighting the significant negative correlation (*p* < 0.01) with the soil pH (Figure 9) could explain its non-detection in the soils of the southern slope (significantly more alkaline than those of the north) (Table 4). This result would agree with that described by Danielson and Davey (1973), who define *T. hamatum* as one of the most widely distributed species in North American acid forest soils. Another aspect to highlight in our study is the significant positive correlation (*p* < 0.05) with the total nitrogen and the organic matter content (Figure 9), which could also contribute to the non-detection in the southern slope soils (with a significantly lower amount of organic matter) (Table 4). These results concur with those described by Migheli et al. (2009) on Sardinia (Italy), where they describe a positive correlation with the content of organic compounds in the soil.

*Trichoderma asperellum* is a species widely used as a BCA against different pathogens, including the different “special forms” (f. sp.) of *F. oxysporum* (Patel and Saraf, 2017; Sehim et al., 2023). Therefore, this species is part of the active ingredient of several microbial fungicides marketed in different countries around the world (Woo et al., 2023). In banana soils, it is cited in different places, such as: India (Thangavelu and Gopi, 2015; Damodaran et al., 2020), Nigeria (Akinyele et al., 2019), and China (Win et al., 2021). Some authors also describe it as an endophytic species of banana (Xia et al., 2011; Thangavelu and Gopi, 2015; Olowe et al., 2022). However, in the Canary Islands, previous works do not describe this species in any of the bioclimatic conditions, while in our study, it was detected as a rare species on the northern slope of healthy and diseased plants (Figures 6–8). The non-detection in the southern soils could be due to the inability to establish in the warmer and drier conditions of this slope of the island. In this sense, similar results were observed by Migheli et al. (2009) on the island of Sardinia (Italy).

*Trichoderma afroharzianum* is an opportunistic environmental species with an outstanding aptitude for biocontrol, plant growth promotion and enzyme production (Chaverri et al., 2016; Xiao et al., 2023). Previous studies do not report this species in the Canary Islands or in the banana rhizosphere in other parts of the world. In this study, this species was detected with a low occurrence frequency (rare species) on the southern slope of both types of plants (healthy and diseased) (Figures 6–8).

*Trichoderma longibrachiatum* has been previously described in banana soils in different parts of the world: Mexico (Hernández-Domínguez et al., 2019), Brazil (Sanó et al., 2022), and Tenerife (Spain) (Ciancio et al., 2022). In our work, this species has been detected as rare (Figure 8) in soils from the southern slope (Figures 6, 7), which agrees with what was previously described by Ciancio et al. (2022).

*Trichoderma* aff. *hortense* was first described in Argentina from horticultural soil samples. However, isolates of this species previously identified under other names (*T. harzianum, T. aureoviride, Hypocrea lixii*) have been described in Japan, Italy, and New Zealand, suggesting a cosmopolitan distribution (Barrera et al., 2021). In our study, the *tef1-α* gene sequences obtained from banana soil isolates from Tenerife were related to the same clade as the *T. hortense* sequences. However, these sequences were not identical to those deposited in the GenBank database (95.6% similarity), therefore, we use the term *affinis* (aff.) to indicate that the OTUs are similar but not identical to the assigned species (accession no. MH253895). According to the Olmstead-Tukey test, this species is classified as rare in the banana crops of Tenerife (Figure 8), with a low number of isolates (3) from the rhizosphere of diseased plants from a single farm on the southern slope (Figures 6, 7).

*Trichoderma gamsii* has been described in Greece, Italy, Spain, Africa, India, and China (Migheli et al., 2009; Ding et al., 2012; Rinu et al., 2014; Jaklitsch and Voglmayr, 2015). Zachow et al. (2009) describes this species in Tenerife in soil/rhizosphere samples from different vegetation zones: Arico (Cardonal/Tabaibal; subtropical, arid) and Buenavista del Norte (Laurisilva/Fayal-Brezal; subtropical, subhumid). In our study, a single isolate was obtained from the rhizosphere of a healthy plant from the southern slope of Tenerife (Guía de Isora) (Figures 6, 7) and to our knowledge, there are no previous records associated with banana crops in other parts of the world. *Trichoderma gamsii* has already demonstrated it can reduce the growth of *Fusarium* head blight in wheat and *F. oxysporum* rot and wilt black gram (*Vigna mungo*) (Alukumbura et al., 2022; Valan-Arasu et al., 2023). Consequently, the ability of *T. gamsii* to control *F. oxysporum* f. sp. *cubense* STR4 under the environmental conditions of the Canary Islands should be analyzed.

*Trichoderma hirsutum* was first described in China in the Shennongjia Natural Reserve in soil samples at 1,200 m.a.s.l. (Chen and Zhuang, 2017) and subsequently in northern Algeria (Sub-humid bioclimate) at very low frequency from the tomato rhizosphere (Benttoumi et al., 2020). In Tenerife, a single isolate was obtained from the rhizosphere of a healthy plant from the northern slope (La Orotava) (Figures 6, 7).

## Conclusion

This work represents the first investigation of the biodiversity of *Trichoderma* species in the soil rhizosphere of the Canary Island banana agroecosystem. The results presented here mainly concern the taxonomic analysis of *Trichoderma* and the distribution of the species in the rhizosphere of healthy and *Foc*-STR4 diseased plants in different bioclimatic conditions of Tenerife (Canary Islands, Spain). The findings demonstrate the biodiversity of *Trichoderma* in banana crops. Many of the species identified (6 out of 12 total) had not been previously described in the Canary Islands (*T. afroharzianum, T. asperellum, T. atrobrunneum, T. guizhouense, T. hamatum*, and *T. hirsutum*) or associated to banana rhizosphere (*T. afroharzianum, T. atrobrunneum, T. gamsi, T. guizhouense, T. hirsutum*, and *T. virens*). Therefore, to the best of our knowledge, this work is the first report of these species in the Canary Islands or in banana ecosystems. In addition, two putative novel species, named *T.* aff. *harzianum* and *T.* aff. *hortense*, were detected. The analysis of species distribution showed that *T. virens* and *T.* aff. *harzianum* were the most abundant (35.89% and 27.48% RA, respectively) (Figure 2) and dominant species in the rhizosphere of banana soils on both slopes of Tenerife (north and south) (Figures 7, 8). While other species were mainly associated with a particular bioclimatic condition: *T. hamatum, T. asperellum*, and *T. hirsutum* were isolated only on the northern slope, and *T. afroharzianum, T. longibrachiatum, T. gamsii*, and *T.* aff. *hortense* on the southern slope (Figures 6, 7). Probably, the species associated with the northern slope are better adapted to lower temperatures and higher humidity, while the species associated with the southern slope are better adapted to higher temperatures and lower humidity. Likewise, the edaphic characteristics could influence species distribution: soils on the southern slope had a significantly higher pH than those on the north, while the relationship of the organic matter was the opposite.

*Trichoderma* species have long been recognized as agents for the control of plant diseases and for their ability to enhance root growth and development, crop productivity, resistance to abiotic stresses, and nutrient uptake and utilization (Berg et al., 2005). Knowledge and understanding of the biodiversity of the rhizosphere microbiota in relation to plant health and the environment is essential for the prevention and treatment of plant diseases (Xue et al., 2015). For this reason, it is recommended to select potential biocontrol agents from native *Trichoderma* isolates obtained under similar conditions (soil or plant parts) to those encountered during pathogen control (Howell, 2003). These isolates will be naturally adapted to the specific environmental conditions of biocontrol (temperature, humidity, nutrient availability, microbiota, etc.) and represent a suitable strategy to overcome problems related to the introduction of exogenous microorganisms (Howell, 2003; Anees et al., 2010). In this context, the analysis of the *Trichoderma* population distribution and the culture collection developed in this study will contribute to the future selection of new isolates or novel native species as BCAs against the most important banana disease in the Canary Islands (*Foc*-STR4). In this sense, our work demonstrates the existence of a high number of *Trichoderma* species with biocontrol potential and that are naturally adapted to the environmental and agronomic conditions of banana cultivation in the Canary Islands. This is in line with the ultimate goal of finding a cost-effective and environmentally friendly alternative for disease control.

## Data availability statement

The datasets generated for this study can be found in the GenBank (https://www.ncbi.nlm.nih.gov/genbank/) accession number (OQ858692 to OQ858800).

## Author contributions

RC-D: Data curation, Formal analysis, Investigation, Software, Visualization, Writing – original draft. PB-L: Data curation, Investigation, Writing – original draft. MJV: Funding acquisition, Resources, Supervision, Writing – review & editing. FL: Formal analysis, Funding acquisition, Methodology, Project administration, Resources, Supervision, Validation, Visualization, Writing – review & editing.

## Footnotes

1
www.icia.es

2
www.isth.info

3
http://www.ncbi.nlm.nih.gov/genbank/
