# Supplementary material for: Biodiversity of Trichoderma species of healthy and Fusarium wilt-infected banana rhizosphere soils in Tenerife (Canary Islands, Spain)
Source: Front Microbiol. 2024 May 10;15:1376602. doi: 10.3389/fmicb.2024.1376602 (PMC11122028; doi:10.3389/fmicb.2024.1376602)
Supplement: Supplementary file 1 [file Data_Sheet_1.pdf]

Supplementary Material

Supplementary Table S1. Climatological data of northern and southern slope of Tenerife (Canary Islands).

	**Temperature** (ºC)			**Humidity** (%)			
**Slope**	Mean	Mean maximum	Mean minimum	Mean	Mean maximum	Mean minimum	**Total radiation** (Wh/m^2 - day)
North	19.7	27.7	13.6	77.6	98.1	43.8	4733.4
South	21.8	30.6	13.5	74.9	99.4	34.3	5345.3

Average data for the last 5 years prior to the sampling period corresponding to the meteorological stations (8 stations) of Cabildo Insular de Tenerife located in the surroundings of the sampled farms. (https://www.agrocabildo.org/agrometeorologia_estaciones.asp)

Supplementary Table S2. *Trichoderma* species recovered from banana rhizosphere soils in Tenerife (Canary Islands), with detailed information on location, isolate code and GenBank accession number of the translation elongation factor 1-α region (*tef1*-α) gene sequences.

**Species**	**Location**	**Farm code**	**Slope**	**Isolate code**	**NCBI GenBank accession number**
Trichoderma aff. *harzianum*	Adeje	AD46	S	AD46-S2-2	OQ858692
Trichoderma aff. *harzianum*	Adeje	AD46	S	AD46-S2-5	OQ858693
Trichoderma aff. *harzianum*	Arona	AR51	S	AR51-S1-2	OQ858694
Trichoderma aff. *harzianum*	Arona	AR51	S	AR51-S2-2	OQ858695
Trichoderma aff. *harzianum*	Arona	AR51	S	AR51-S2-4	OQ858696
Trichoderma aff. *harzianum*	Arona	AR51	S	AR51-S3-2	OQ858697
Trichoderma aff. *harzianum*	Arona	AR51	S	AR51-S4-3	OQ858698
Trichoderma aff. *harzianum*	Arona	AR51	S	AR51-S4-4	OQ858699
Trichoderma aff. *harzianum*	Arona	AR51	S	AR51-S4-5	OQ858700
Trichoderma aff. *harzianum*	Arona	AR51	S	AR51-S5-1	OQ858701
Trichoderma aff. *harzianum*	Arona	AR51	S	AR51-S5-4	OQ858702
Trichoderma aff. *harzianum*	Arona	AR51	S	AR51-S6-1	OQ858703
Trichoderma aff. *harzianum*	Arona	AR68	S	AR68-S6-3	OQ858704
Trichoderma aff. *harzianum*	Arona	AR68	S	AR68-S6-5	OQ858705
Trichoderma aff. *harzianum*	Buenavista del Norte	BN47	N	BN47-S6-2	OQ858708
Trichoderma aff. *harzianum*	Los Silos	BN4	N	BN4-S2-3	OQ858706

Trichoderma aff. *harzianum*	Los Silos	BN4	N	BN4-S6-6	OQ858707
Trichoderma aff. *harzianum*	Cueva del Polvo	CPE	S	CPE-S2-5	OQ858709
Trichoderma aff. *harzianum*	Güímar	GU1	S	GU1-S4-1	OQ858710
Trichoderma aff. *harzianum*	Icod de los Vinos	IV37	N	IV37-S2-5	OQ858711
Trichoderma aff. *harzianum*	Icod de los Vinos	IV37	N	IV37-S3-2	OQ858712
Trichoderma aff. *harzianum*	Icod de los Vinos	IV37	N	IV37-S3-5	OQ858713
Trichoderma aff. *harzianum*	Icod de los Vinos	IV37	N	IV37-S5-1	OQ858714
Trichoderma aff. *harzianum*	Icod de los Vinos	IV37	N	IV37-S5-2	OQ858715
Trichoderma aff. *harzianum*	Icod de los Vinos	IV37	N	IV37-S5-3	OQ858716
Trichoderma aff. *harzianum*	Icod de los Vinos	IV37	N	IV37-S5-4	OQ858717
Trichoderma aff. *harzianum*	San C. de la Laguna	PH1	N	PH1-S6-2	OQ858718
Trichoderma aff. *harzianum*	S. C. de la Laguna	PH1	N	PH1-S6-3	OQ858719
Trichoderma aff. *harzianum*	S. C. de la Laguna	PH1	N	PH1-S6-4	OQ858720
Trichoderma aff. *harzianum*	S. C. de la Laguna	TV26	N	TV26-S1-2	OQ858721
Trichoderma aff. *harzianum*	La Orotava	VO78	N	VO78-S5-2	OQ858722
Trichoderma aff. *hortense*	Adeje	AD46	S	AD46-S3-2	OQ858798
Trichoderma aff. *hortense*	Adeje	AD46	S	AD46-S3-3	OQ858799
Trichoderma aff. *hortense*	Adeje	AD46	S	AD46-S3-5	OQ858800
Trichoderma afroharzianum	Arona	AR51	S	AR51-S1-3	OQ858734
Trichoderma afroharzianum	Arona	AR51	S	AR51-S6-2	OQ858735
Trichoderma asperellum	S. C. de la Laguna	PH1	N	PH1-S5-2	OQ858793
Trichoderma asperellum	La Orotava	VO78	N	VO78-S4-7	OQ858792
Trichoderma atrobrunneum	Buenavista del Norte	BN47	N	BN47-S2-2	OQ858781
Trichoderma atrobrunneum	Buenavista del Norte	BN47	N	BN47-S2-3	OQ858782
Trichoderma atrobrunneum	Buenavista del Norte	BN47	N	BN47-S2-5	OQ858783
Trichoderma atrobrunneum	Buenavista del Norte	BN47	N	BN47-S4-2	OQ858784
Trichoderma atrobrunneum	Los Silos	BN4	N	BN4-S1-4	OQ858778
Trichoderma atrobrunneum	Los Silos	BN4	N	BN4-S2-5	OQ858779
Trichoderma atrobrunneum	Los Silos	BN4	N	BN4-S4-2	OQ858780
Trichoderma atrobrunneum	S. C. de la Laguna	PH1	N	PH1-S1-2	OQ858785
Trichoderma atrobrunneum	S. C. de la Laguna	PH1	N	PH1-S1-3	OQ858786
Trichoderma atrobrunneum	S. C. de la Laguna	PH1	N	PH1-S6-1	OQ858787
Trichoderma atrobrunneum	San Miguel de Abona	SM109	S	R-SM109-S2-2	OQ858788
Trichoderma gamsii	Cueva del polvo	CPE	S	CPE-S2-1	OQ858791
Trichoderma guizhouense	Arona	AR68	S	AR68-S3-3	OQ858736
Trichoderma guizhouense	Los Silos	BN4	N	BN4-S4-3	OQ858737
Trichoderma guizhouense	Los Silos	BN4	N	BN4-S5-9	OQ858738
Trichoderma guizhouense	Icod de los Vinos	IV37	N	R-IV37-S4-4A	OQ858740
Trichoderma guizhouense	Icod de los Vinos	IV72	N	IV72-S3-3	OQ858739
Trichoderma guizhouense	San Miguel de Abona	SM109	S	SM109-S2-5	OQ858741
Trichoderma guizhouense	La Orotava	VO78	N	VO78-S4-10	OQ858742
Trichoderma hamatum	Icod de los Vinos	IV72	N	IV72-S2-1	OQ858797

Trichoderma hamatum	Icod de los Vinos	IV72	N	IV72-S5-2	OQ858796
Trichoderma hamatum	Icod de los Vinos	IV72	N	IV72-S6-2	OQ858795
Trichoderma hamatum	La Orotava	VO64	N	VO64-S4-4	OQ858794
Trichoderma harzianum	Adeje	AD46	S	AD46-S2-3	OQ858723
Trichoderma harzianum	Adeje	AD46	S	AD46-S6-3	OQ858724
Trichoderma harzianum	Adeje	AD46	S	AD46-S6-5	OQ858725
Trichoderma harzianum	Adeje	AD46	S	AD46-S6-6	OQ858726
Trichoderma harzianum	Arona	AR68	S	AR68-S3-6	OQ858727
Trichoderma harzianum	Buenavista del Norte	BN47	N	BN47-S4-3	OQ858728
Trichoderma harzianum	Icod de los Vinos	IV37	N	R-IV37-S2-3A	OQ858731
Trichoderma harzianum	Icod de los Vinos	IV72	N	IV72-S3-1	OQ858729
Trichoderma harzianum	Icod de los Vinos	IV72	N	R-IV72-S4-3B	OQ858730
Trichoderma harzianum	San Miguel de Abona	SM109	S	R-SM109-S1-5	OQ858732
Trichoderma hirsutum	La Orotava	VO78	N	VO78-S4-3	OQ858733
Trichoderma longibrachiatum	San Miguel de Abona	SM109	S	R-SM109-S2-4	OQ858789
Trichoderma longibrachiatum	San Miguel de Abona	SM109	S	R-SM109-S3-5	OQ858790
Trichoderma virens	Adeje	AD46	S	AD46-S2-4	OQ858743
Trichoderma virens	Arona	AR51	S	AR51-S5-3	OQ858744
Trichoderma virens	Arona	AR51	S	AR51-S6-6	OQ858745
Trichoderma virens	Arona	AR68	S	AR68-S1-1	OQ858746
Trichoderma virens	Arona	AR68	S	AR68-S1-2	OQ858747
Trichoderma virens	Arona	AR68	S	AR68-S1-3	OQ858748
Trichoderma virens	Arona	AR68	S	AR68-S1-4	OQ858749
Trichoderma virens	Arona	AR68	S	AR68-S1-5	OQ858750
Trichoderma virens	Arona	AR68	S	AR68-S6-4	OQ858751
Trichoderma virens	Arona	AR68	S	R-AR68-S6-6	OQ858767
Trichoderma virens	Buenavista del Norte	BN47	N	BN47-S5-5	OQ858756
Trichoderma virens	Buenavista del Norte	BN47	N	BN47-S5-6	OQ858757
Trichoderma virens	Buenavista del Norte	BN47	N	BN47-S5-7	OQ858758
Trichoderma virens	Los Silos	BN4	N	BN4-S1-1	OQ858752
Trichoderma virens	Los Silos	BN4	N	BN4-S5-1	OQ858753
Trichoderma virens	Los Silos	BN4	N	BN4-S5-2	OQ858754
Trichoderma virens	Los Silos	BN4	N	BN4-S5-6	OQ858755
Trichoderma virens	Cueva del polvo	CPE	S	CPE-S2-3	OQ858759
Trichoderma virens	Cueva del polvo	CPE	S	CPE-S5-1	OQ858760
Trichoderma virens	Cueva del polvo	CPE	S	CPE-S5-2	OQ858761
Trichoderma virens	Icod de los Vinos	IV37	N	IV37-S1-3	OQ858762
Trichoderma virens	Icod de los Vinos	IV37	N	IV37-S3-3	OQ858763
Trichoderma virens	Icod de los Vinos	IV37	N	IV37-S6-1	OQ858764
Trichoderma virens	Icod de los Vinos	IV37	N	R-IV37-S2-4B	OQ858768
Trichoderma virens	Icod de los Vinos	IV72	N	IV72-S1-4	OQ858765
Trichoderma virens	Icod de los Vinos	IV72	N	IV72-S4-5	OQ858766
Trichoderma virens	San Miguel de Abona	SM109	S	SM109-S3-2	OQ858770

Trichoderma virens	San Miguel de Abona	SM109	S	SM109-S3-3	OQ858771
Trichoderma virens	San Miguel de Abona	SM109	S	R-SM109-S1-4	OQ858769
Trichoderma virens	La Orotava	VO78	N	VO78-S2-3	OQ858772
Trichoderma virens	La Orotava	VO78	N	VO78-S4-1	OQ858773
Trichoderma virens	La Orotava	VO78	N	VO78-S4-4	OQ858774
Trichoderma virens	La Orotava	VO78	N	VO78-S4-5	OQ858775
Trichoderma virens	La Orotava	VO78	N	VO78-S5-1	OQ858776
Trichoderma virens	La Orotava	VO78	N	VO78-S5-3	OQ858777

N: north. S: south. S. C. de La Laguna: San Cristóbal de la Laguna

Supplementary Table S3. Species of *Trichoderma* and NCBI GenBank accessions numbers of the translation elongation factor 1-α (*tef1*-α) used in this study for phylogenetic analyses.

		**GenBank accession No.**
**Species**	**Strain ID**	***tef1*-α**
Protocrea illinoensis	GJS 94-54	EU703904
Protocrea pallida	CBS 299.78 ^{T}	EU703900
T. asperellum	ACCC 32725	MF049065
T. aethiopicum	PPRC H5	EU401616
T. afarasin	DIS 314F^{T}	FJ463400
T. afroharzianum	GJS 04-186 ^{T}	FJ463301
T. afroharzianum	HZA3	MK850825
T. aggressivum	DAOM 222156 ^{T}	AF348098
T. aggressivum f. sp. *europaeum*	CBS 689.94 ^{T}	KP008996
T. alni	CBS 120633	EU498312
T. amazonicum	IB 50	HM142376
T. andinense	GJS 90-140 ^{T}	AY956321
T. asperelloides	GJS 04-116	GU248412
T. atrobrunneum	GJS 05-101	FJ463392
T. atrobrunneum	S414	KJ665393
T. atroviride	GJS 98-134	AF456887
T. austroindianum	BAFC 3583^{T}	MH352421
T. bannaense	HMAS 248840 ^{T}	KY688037
T. britdaniae	K 89878 ^{T}	JQ685865
T. brunneoviride	CBS 121130	EU498316

T. camerunense	GJS 99-230 T	AF348107
T. camerunense	GJS 99-231	AF348108
T. capillare	CPK. 2883 T	JN182283
T. caribbaeum	CBS 119093 T	KJ665443
T. catoptron	GJS 02-76	AY737726
T. christiani	CBS 132572 T	KJ665439
T. chromospermum	GJS 94-67 T	AY737728
T. cinnamomeum	GJS 97-237	AY737732
T. citrinoviride	CBS 258.85 T	AY865637
T. corneum	GJS 97-75 T	AY937431
T. crassum	DAOM 164916 T	EU280048
T. eijii	CBS 133190 T	JX684011
T. endophyticum	Dis 217A T	FJ463319
T. evansii	CBS 123079 T	EU883566
T. flagellatum	PPRC-ET58 T	FJ763184
T. flaviconidium	GJS 99-49	DQ020001
T. gamsii	GJS 04-09	DQ307541
T. gamsii	S496	KJ665494
T. ghanense	GJS 95-137 T	AY937423
T. gillesii	GJS 00-72 T	JN175583
T. gracile	GJS 10-263 T	JN175598
T. guizhouense	CBS 131803- HGUP0038	JN215484
T. guizhouense	GJS 97-28 T	AY937440
T. guizhouense	GJS 08-102	MG797484
T. hamatum	S397	JN715614
T. hamatum	DAOM 167057 T	AF456911
T. harzianum	CBS 226.95 T	AF348101
T. harzianum	CBS 227.95	AF348100
T. hirsutum	HMAS 248834 T	KY688029
T. hirsutum	HMAS 248859	KY688030
T. hortense	BMCC:LU994	KJ871185
T. hortense	DAOM 230830	AY605786
T. hortense	GJS 04-70	FJ463352
T. hortense	BAFC 4291 GJS08-116 T	MH253895
T. hortense	NR 6931	AF348104
T. inhamatum	CBS 273.78 T	AF348099
T. konilangbra	CPK. 132	JN258681
T. koningiopsis	GJS 93-20 T	DQ284966
T. kunmingense	YMF1.02659 T	KJ742802
T. lentiforme	Dis 253B	FJ851875
T. lentiforme	Dis 110A T	FJ851872
T. lixii	G.J.S.97-96 T	AF443938
T. longibrachiatum	CBS 816.68 T	EU401591

T. neokoningii	CBS 120070 T	KJ665620
T. neorufoides	CBS 119506 T	FJ860657
T. neorufum	CBS 119498 T	FJ860653
T. neotropicale	LA11T	HQ022771
T. neotropicale	T51	FJ967825
T. novae-zelandiae	GJS 81-265 T	AY937448
T. ochroleucum	CBS 119502	FJ860659
T. orientale	GJS 88-81 T	EU401581
T. ovalisporum	DAOM 232077 T	KJ871200
T. parareesei	TUB F-1066 CBS 125925 T	GQ354353
T. paratroviride	CBS 136489 T	KJ665627
T. paraviridescens	CBS 119321 T	DQ672610
T. patella	G.J.S. 91-141	KJ665630
T. paucisporum	GJS 01-13 T	DQ109540
T. pezizoides	GJS 01-257	AY937438
T. pinnatum	GJS 04-100 T	JN175571
T. pleuroti	CBS 124387	HM142382
T. pleuroticola	CBS 124383 T	HM142381
T. pollinicola	LC 11682	MF939621
T. pseudokoningii	DAOM 167678 T	KJ713204
T. pubescens	DAOM 166162 T	AY750887
T. pyramidale	CBS 135574 T	KJ665699
T. reesei	GJS 00-89	JN175599
T. rifaii	Dis 337f T	FJ463321
T. rifaii	Dis 355B	FJ463324
T. saturnisporopsis	C.P.K. 1356 T	JN182281
T. saturnisporum	ATCC 28023	JN388897
T. silvae-virgineae	CBS 120922	FJ860696
T. simmonsii	G.J.S.91-138 T	AF443935
T. sinense	DAOM 230004 T	AY750889
T. solani	GJS 08-81 T	JN175597
T. stilbohypoxyli	CBS 992.97 T	DQ109546
T. stramineum	GJS 02-84	AY737746
T. stromaticum	CQSQ1032	JQ040422
T. stromaticum	GJS 97-180 T	HQ342166
T. subviride	HMAS 273761	KU529131
T. syagri	BAFC 4357T	MG822711
T. tawa	GJS 97-174 T	FJ463313
T. theobromicola	Dis 85f	EU856321
T. valdunense	CBS 120923	FJ860717
T. virens	DAOM 167652T	AY750891
T. virens	LZ011	ON010787

T. virens	HZA14	MK850836
T. virens	GJS 01-287	AY750894
T. virens	EEKC6	OP688487
T. viride	CBS 119325 **T**	DQ672615
T. voglmayrii	CBS 117711 **T**	DQ086146
T. xanthum	TC714	MF371226
T. yunnanense	CBS 121219 **T**	GU198243
T. zeloharzianum	YMF1.00268 **T**	MH183181

(T = type strain).
